# Opioid and dopamine mediation of gambling responses in recreational gamblers

**DOI:** 10.3389/fnbeh.2013.00147

**Published:** 2013-10-14

**Authors:** Martin Zack

**Affiliations:** Centre for Addiction and Mental Health, Neuroscience ResearchToronto, ON, Canada

**Keywords:** gambling, opioid, dopamine, cognition, motivation, emotion

Cognitions play an important role in addictive behavior. This may be especially true for “behavioral addictions,” like pathological gambling, where reinforcement derives from environmental events whose value is, for the most part, learned. The study by Porchet and colleagues examines the roles of dopamine and the endogenous opioids in response to tasks designed to evoke gambling-related cognitive distortions in recreational gamblers. The investigators report that the dopamine D2 receptor antagonist, haloperidol had little effect on subjective responses to near-misses (outcomes that closely approximate wins) but slightly enhanced physiological response to these stimuli. In contrast, the mixed opioid receptor antagonist, naltrexone increased physiological reactivity to these stimuli and also increased subjective confidence to predict future outcomes following a winning streak on a roulette task. The findings for haloperidol are consistent with the increased physiological response and lack of subjective effects of this drug on response to gambling activity previously seen in healthy individuals. The findings for naltrexone are counterintuitive, given that naltrexone and the opioid antagonist nalmefene have proven effective in curbing urges to gamble in pathological gamblers. Although not entirely predicted, the results confirm that, like drugs of abuse, gambling activity reliably engages the dopamine and opioid systems. Together with other evidence, they also indirectly suggest that recreational gamblers may respond differently to drug manipulations than pathological gamblers due to functional differences in the brains of these two populations. Whereas the effects in recreational gamblers reflect a perturbation from homeostatic baseline function, the increase in dopamine cell firing induced by haloperidol and increase in stress axis responding induced by naltrexone may act to restore or mitigate deviations from normal brain function that represent the new baseline or “allostatic” brain state of the pathological gambler. Replication of this experiment in pathological gamblers would be a valuable complement to this important study.

